# Supplementary material for: DNA methylation outlier burden, health, and ageing in Generation Scotland and the Lothian Birth Cohorts of 1921 and 1936
Source: Clin Epigenetics. 2020 Mar 26;12:49. doi: 10.1186/s13148-020-00838-0 (PMC7098133; doi:10.1186/s13148-020-00838-0)
Supplement: Supplementary file 2 — Additional file 2: Supplementary Figures 1–5. and Supplementary Notes 1 and 2. [file 13148_2020_838_MOESM2_ESM.docx]

**Supplementary Note 1**

- 1. **DNA methylation quality control in Generation Scotland set 1**

Genome-wide DNA methylation in whole blood was measured using the Illumina Infinium HumanMethylationEPIC BeadChip. Quality control was carried out in R (1). Log median intensity of methylated versus unmethylated signal per array was plotted using *ShinyMethyl* (2), and outliers were excluded manually upon visual inspection. The *WateRmelon* package (3) was used to remove samples in which >1% of probes had a detection p-value >0.05, and probes in which >5% of samples had a detection p-value >0.05. In addition, probes with a beadcount <3 in more than 5% of samples and samples in which methylation-predicted sex (based on the difference between the median copy number intensity for the Y chromosome and the median copy number intensity for the X chromosome) did not match recorded sex were excluded using *ShinyMethyl*. *ShinyMethyl* was also used to plot the output from control probes and these plots were also visually inspected for outliers. No additional outliers were identified (having already removed the samples that were outliers in the log median methylated vs. unmethylated signal plot). Finally, multi-dimensional scaling plots were inspected for additional sample outliers but no samples were excluded on the basis of this.

- 1. **DNA methylation quality control in Generation Scotland set 2**

Genome-wide DNA methylation in whole blood was measured using the Illumina Infinium HumanMethylationEPIC BeadChip. In Generation Scotland set 2, quality control was initially carried out using the *meffil* package (4). Samples were excluded if self-reported sex did not match methylation-predicted sex; if >1% CpGs had a detection *p*-value >0.05; if they showed evidence of dye bias or were outliers for the bisulphite conversion control probes; or if they had a median methylated signal intensity more than three standard deviations lower than expected. Following the removal of the poor-performing samples detected by *meffil*, *minfi* (5) was used to read data into R for a second round of quality control performed using *shinyMethyl* (2) as described for set 1. Following the exclusion of samples identified by the steps above, multi-dimensional scaling plots were inspected for additional sample outliers and these were excluded too. Finally, poor-performing probes with a beadcount <3 in >5% of samples or more than >5% samples of detection *p*-value >0.05 were identified and removed using *meffil.*

- 1. **DNA methylation quality control in the Lothian Birth Cohorts of 1921 and 1936**

Briefly, data were background corrected and normalised using internal controls. Samples of low quality (bisulphite conversion, staining signal, inadequate hybridisation or nucleotide extension) were removed. Probes on the X and Y chromosomes, as well as probes with a detection rate <95% at p<0.01 were excluded. In addition, samples with a low call rate <450,000 probes at p<0.01, samples for which sex predicted by DNA-methylation did not match reported sex, as well as samples showing a poor match between SNP control probes and genotype were excluded. More detail is available elsewhere (6,7).

**Supplementary Note 2**

**2.1. Covariates in Generation Scotland set 1 and set 2**

**2.1.1. Cell proportions**

Proportions of granulocytes, natural killer cells, B-cells, CD4+ T-cells and CD8+ T-cells were estimated using the Houseman method (8) as implemented in the *estimateCellCounts* function in *minfi* (5).

**2.1.2. Technical factors**

In addition to set, batch was included as a technical factor in the analysis of Generation Scotland data. Methylation profiling in set 1 and set 2 was carried out in 31 batches each.

**2.1.3. Health and lifestyle**

Prior to a visit to the clinic, pre-clinical questionnaires were administered to Generation Scotland participants. Among others, participants completed the Pre-Clinical Questionnaire (PCQ) including questions on physical health and family history of disease as well as lifestyle factors. Physical and biochemical measures were collected at the clinic visit.

A measure of cancer diagnosis (Yes/No) was created by combining self-reports of four types of cancer (breast, bowel, lung and prostate cancer). In addition, an ever vs. never smoking variable and a log10-transformed pack-years variable (calculated by multiplying the number of packs smoked per day by the number of years of smoking) and a log10-transformed version of body mass index (BMI, calculated from height and weight in kg/m^2^) were included as covariates. Variables were log10-transformed in order to minimise positive skew. Missing values in the ever-smoking variable were kept as an ‘unknown’ category. For 17 individuals who had no information on smoking status, missing pack-years values were median imputed, which led to the assignment of a zero value.

**2.2. Covariates in the Lothian Birth Cohorts**

**2.2.1. Cell proportions**

As in the Generation Scotland data, cell proportions (granulocytes, natural killer cells, B-cells, CD4+ T-cells and CD8+ T-cells) were estimated using the Houseman method (8).

**2.2.2. Technical factors**

Methylation profiling in the LBC1921 was carried out in one batch on 7 dates, using 11 plates and 76 arrays. Methylation profiling in the LBC1936 was carried out in three batches on 13 dates, using 41 plates and 309 arrays.

**2.2.3. Health and lifestyle**

At each wave of data collection, participants completed questionnaires providing information on health and lifestyle. Physical measures such as height and weight were measured by trained research nurses as part of a clinical assessment.

A binary ever-never smoking variable, a log10-transformed smoking pack-years variable, a binary self-reported cancer diagnosis (Yes/No) variable and a log10-transformed measure of BMI were used as covariates. The pack-years variable for each individual was calculated based on reports of the number of cigarettes smoked, age started and age stopped which had been collected in wave 1 of the data collection. All other variables were longitudinal measures and available for each wave of data collection.

**Supplementary Tables (Additional File 1, Excel)**

**Supplementary Table 1** Descriptive Statistics. Sample numbers (N) alongside their proportion in the entire sample (in %) are reported for categorical variables. Mean (M) and Standard Deviation (SD) values are reported for continuous variables.

**Supplementary Table 2** The association between DNA methylation outlier burden (log10 transformed) and age from models with basic (A) to full (C) adjustments. Standardised and raw regression coefficients, standard errors of raw regression coefficients (SE) and p-values are reported for models of DNAm outlier burden (log10 transformed) and age from models with different adjustments. Associations between outlier burden and age are cross-sectional in Generation Scotland and longitudinal in the Lothian Birth Cohorts. The standardised coefficients give change in standard deviations of log10(outlier burden) per 1-year change in age.

**Supplementary Table 3** Multivariate regression analysis of DNAm outlier burden in Generation Scotland, the LBC1921 and the LBC1936. Standardised and raw regression coefficients, standard errors of raw regression coefficients (SE) and p-values are shown for fixed effects from fully adjusted models of *log10(outlier burden) ~ age + covariates*. Associations are cross-sectional in Generation Scotland and longitudinal in the Lothian Birth Cohorts. All continuous variables were transformed to z-scores prior to running the model in order to standardise coefficients for ease of interpretation. The standardised coefficients give change in standard deviations of log10(outlier burden).

**Supplementary Table 4** The association between DNA methylation outlier burden (log10 transformed) calculated by the alternative definition in Generation Scotland and age from models with basic to full adjustments [A] as well as with all fixed-effect covariates in the fully adjusted model [B]. DNAm outlier burden was calculated in age deciles. All continuous variables were transformed to z-scores prior to running the model in order to standardise coefficients for ease of interpretation. Standardised and raw regression coefficients, standard errors of raw regression coefficients (SE) and p-values are shown. The standardised coefficients give change in standard deviations of log10(outlier burden).

**Supplementary Table 5** Cross-sectional associations between DNAm outlier burden and self-reported disease in Generation Scotland. Odds ratios (OR), 95% Confidence Intervals (95% CI), p-values, sample sizes and prevalence numbers from logistic regression models of self-reported disease and a scaled version of log10(outlier burden) in Generation Scotland with basic [A] and full [B] adjustments as detailed in the main text. Odds ratios are given per standard deviation increase in log10(outlier burden). The cancer variable was created by combining reports of 4 types of cancer with individually low prevalence rates: Breast, bowel, lung and prostate cancer.

**Supplementary Table 6** Cross-sectional associations between DNAm outlier burden and family history of disease in Generation Scotland. Odds ratios (OR), 95% Confidence Intervals (95% CI), p-values, sample sizes and prevalence numbers from logistic regression models of family history of disease in mother or father and a scaled version of log10(outlier burden) in Generation Scotland with basic [A] and full [B] adjustments as detailed in the main text. Odds ratios are given per standard deviation increase in log10(outlier burden).

**Supplementary Table 7** DNAm outlier burden and survival in the Lothian Birth Cohorts. Cox models of survival and a scaled version of log10(outlier burden) with basic (A) and full (B) adjustments as detailed in the main text. Hazard ratios are given per standard deviation increase in log10(outlier burden).

**Supplementary Table 8** Sample numbers by wave of data collection and processing batch in the LBC1936. All LBC1921 samples were processed in set 1.


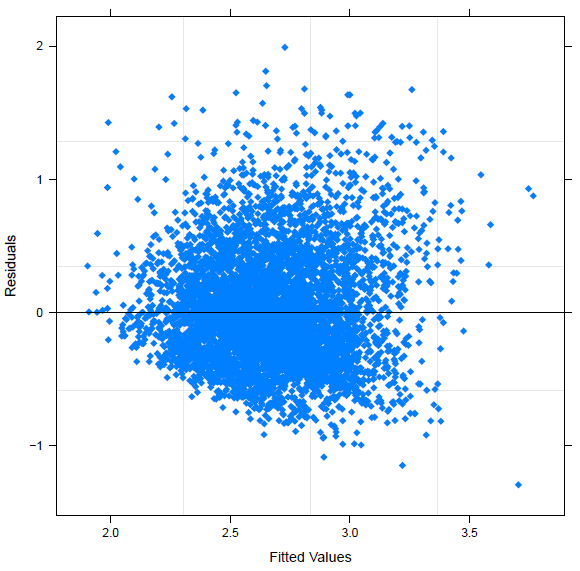


**[C]**

**[A]**


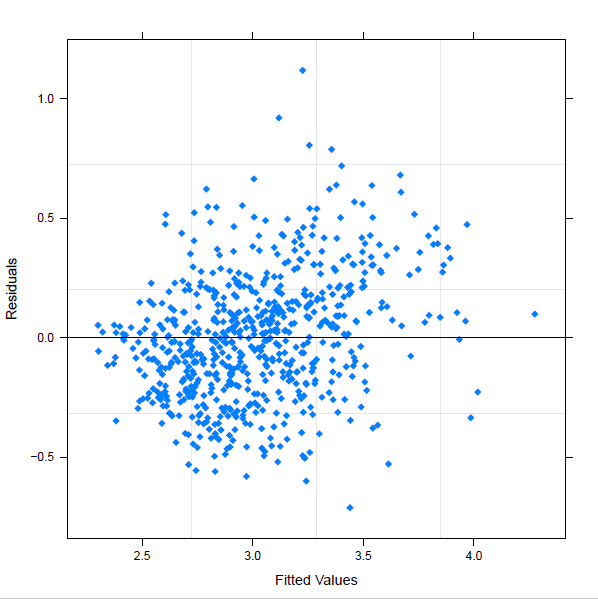

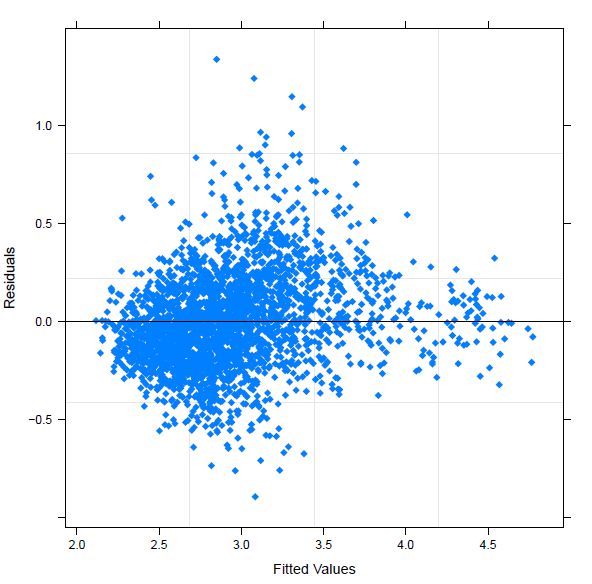


**[C]**

**[B]**

**Supplementary Figure 1** Model diagnostics plotting residuals (y-axis) against fitted values (x-axis) for the fully adjusted models of *log10(outlier burden) ~ age* trajectories in Generation Scotland [A], the LBC1921 [B] and the LBC1936 [C].


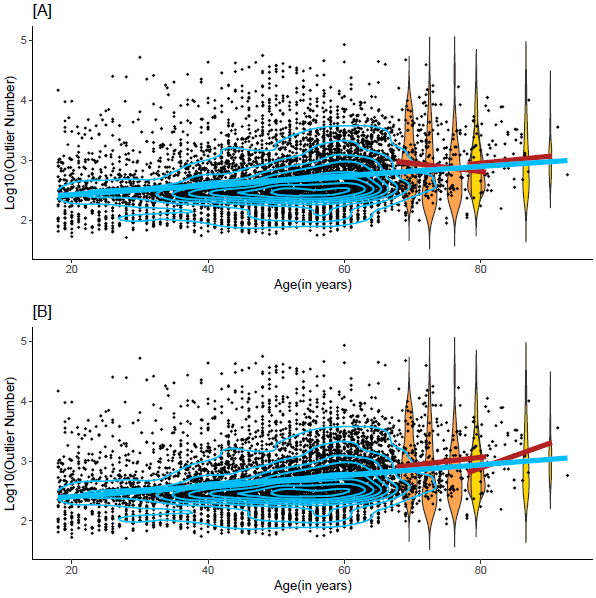


**Supplementary Figure 2** Distribution of log10 (outlier burden) in Generation Scotland (black, blue contour shapes indicating data density) and in the four LBC1936 waves (orange) and three LBC1921 waves (yellow), subset to those individuals with complete observations in all waves (63 individuals in the LBC1921 and 337 individuals in the LBC1936). Panel A shows linear regression lines in Generation Scotland (blue) and in the LBC1921 and LBC1936 (red) to model the association of *log10(outlier burden) ~ age*. Panel B shows fitted values for the regression of *log10(outlier burden) ~ age* with random factor batch and cell proportions fit to the mean.


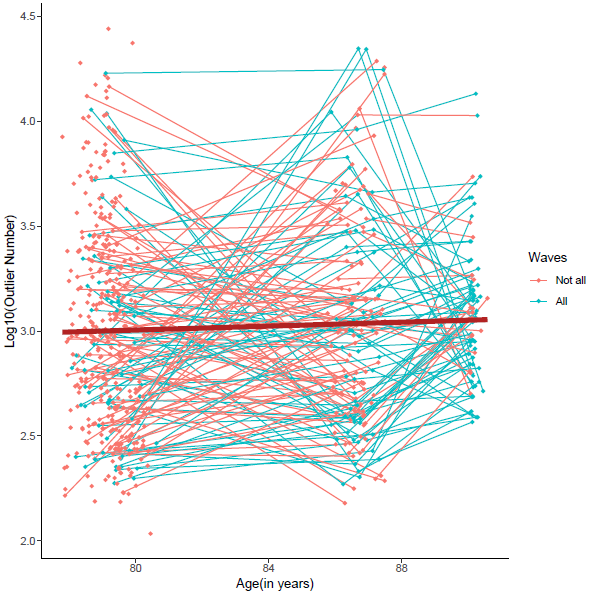


**Supplementary Figure 3** Longitudinal change in log10(outlier burden) in individuals in the LBC1921. Trajectories of individuals with complete observations are shown in blue, those of individuals for whom observations were not available in all waves are shown in red. The linear regression line of *log10(outlier burden) ~ age* is shown in dark red.


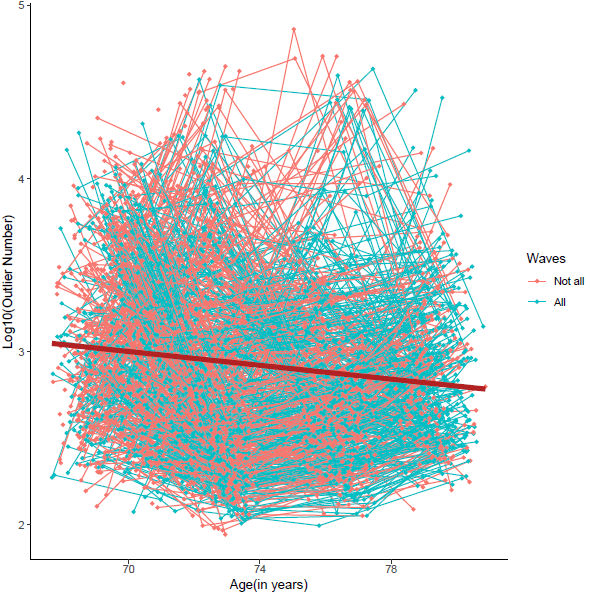


**Supplementary Figure 4** Longitudinal change in log10(outlier burden) in individuals in the LBC1936. Trajectories of individuals with complete observations are shown in blue, those of individuals for whom observations were not available in all waves are shown in red. The linear regression line of *log10(outlier burden) ~ age* is shown in dark red.


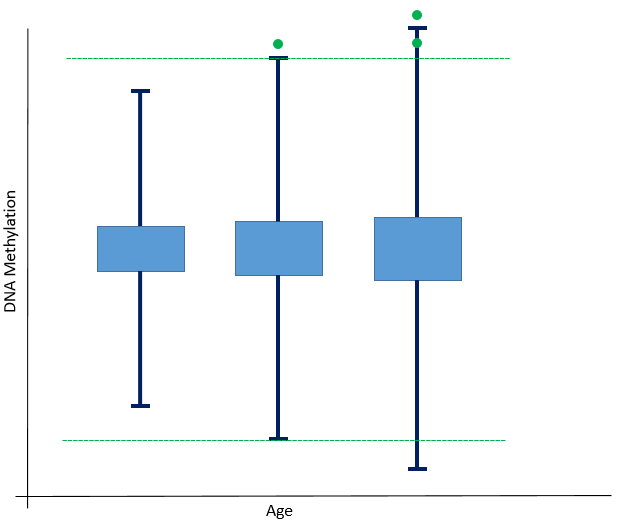
**A**


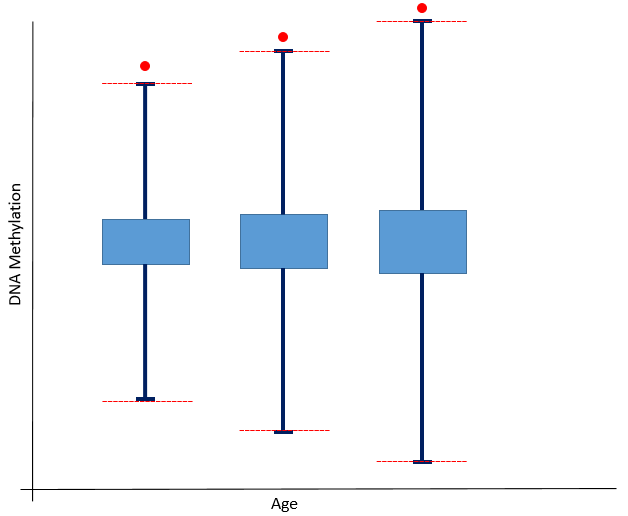
 **B**

**Supplementary Figure 5** Figure illustrating the principle behind epigenetic drift and DNA methylation outlier burden. Boxplots depicting DNA methylation level variability at a (fictional) CpG site with age. The lower and upper border of the box represent the lower and upper quartile of the distribution. The whiskers show three interquartile ranges (IQR) from the upper and lower quartile. The younger age group (on the left) show less variability in methylation levels than the intermediate (middle) and older age group (right). When outliers are defined based on the mean IQR in the entire sample as done by Gentilini and colleagues (2015), outlier burden seems to increase with age [A]. However, when outliers are defined within each age group, outlier burden stays constant across age groups [B].

**References**

1. Core R Team. R a language environment for statistical computing. 2015.

2. Fortin J-P, Fertig E, Hansen K. shinyMethyl: interactive quality control of Illumina 450k DNA methylation arrays in R [v2]. 2014.

3. Wong CCY, Pidsley R, Schalkwyk LC. The wateRmelon Package. 2013.

4. Min JL, Hemani G, Davey Smith G, Relton C, Suderman M. Meffil: efficient normalization and analysis of very large DNA methylation datasets. Bioinformatics. 2018;34(23):3983–9.

5. Aryee MJ, Jaffe AE, Corrada-Bravo H, Ladd-Acosta C, Feinberg AP, Hansen KD, et al. Minfi: a flexible and comprehensive Bioconductor package for the analysis of Infinium DNA methylation microarrays. Bioinformatics. 2014;30(10):1363–9.

6. Shah S, McRae AF, Marioni RE, Harris SE, Gibson J, Henders AK, et al. Genetic and environmental exposures constrain epigenetic drift over the human life course. Genome Res. 2014;24(11):1725–33.

7. Zhang Q, Marioni RE, Robinson MR, Higham J, Sproul D, Wray NR, et al. Genotype effects contribute to variation in longitudinal methylome patterns in older people. Genome Med. 2018;10(1):75.

8. Houseman EA, Accomando WP, Koestler DC, Christensen BC, Marsit CJ, Nelson HH, et al. DNA methylation arrays as surrogate measures of cell mixture distribution. BMC Bioinformatics. 2012;13(1):86.
